# Measuring orthographic transparency and morphological-syllabic complexity in alphabetic orthographies: a narrative review

**DOI:** 10.1007/s11145-017-9741-5

**Published:** 2017-04-17

**Authors:** Elisabeth Borleffs, Ben A. M. Maassen, Heikki Lyytinen, Frans Zwarts

**Affiliations:** 1 Center for Language and Cognition Groningen (CLCG), School of Behavioral and Cognitive Neurosciences (BCN), University of Groningen, P.O. Box 716, 9700 AS Groningen, The Netherlands; 2 Department of Psychology, University of Jyväskylä, P.O. Box 35, 40014 Jyväskylä, Finland; 3 Niilo Mäki Institute, Jyväskylä, Finland

**Keywords:** Orthographic transparency, Morphological complexity, Syllabic complexity, Measures

## Abstract

This narrative review discusses quantitative indices measuring differences between alphabetic languages that are related to the process of word recognition. The specific orthography that a child is acquiring has been identified as a central element influencing reading acquisition and dyslexia. However, the development of reliable metrics to measure differences between language scripts hasn’t received much attention so far. This paper therefore reviews metrics proposed in the literature for quantifying orthographic transparency, syllabic complexity, and morphological complexity of alphabetic languages. The review included searches of Web of Science, PubMed, PsychInfo, Google Scholar, and various online sources. Search terms pertained to orthographic transparency, morphological complexity, and syllabic complexity in relation to reading acquisition, and dyslexia. Although the predictive value of these metrics is promising, more research is needed to validate the value of the metrics discussed and to understand the ‘developmental footprint’ of orthographic transparency, morphological complexity, and syllabic complexity in the lexical organization and processing strategies.

## Introduction

Regardless of which alphabetic orthography is being acquired, the beginning reader essentially needs to learn to associate letters with sounds in order to access whole-word phonological representations of known words (Grainger & Ziegler, 2011). After deliberate practice and once lexical representations of words have been established in the reader’s memory, a skilled reader no longer needs to rely on phonics when coming across the same word again; reading has become a fast and highly efficient word recognition process (Sprenger-Charolles & Colé, 2003). The specific orthography that a child is acquiring has been identified as a central environmental factor influencing reading acquisition and dyslexia (for a review, see Ziegler & Goswami, 2005). Characteristics of the specific orthography that needs to be learned shape the phonological recoding and reading strategies that are developed for reading. However, the development and availability of metrics to compare orthographic characteristics between languages related to word recognition has received little attention so far. Detailed knowledge of differences between orthographies and metrics to measure these differences would provide a stepping stone in the development of language-specific reading instructions and interventions. Therefore, the aim of this narrative review is to examine several quantitative indices measuring differences related to the process of word recognition in alphabetic languages, with special attention to studies that propose various measures of different granularities at which readers crack the orthographic code to identify written words. We present measures of orthographic transparency, syllabic complexity, and morphological complexity. Additionally, we discuss some suggestions for future studies in this domain.

Research has suggested for transparent orthographies with highly regular grapheme-phoneme correspondences to be more easily acquired than complex and opaque orthographies with a high proportion of irregular and inconsistent spellings (e.g., Aro & Wimmer, 2003; Seymour, Aro, & Erskine, 2003). It has even been postulated that children at the lower end of the reading-ability spectrum show less severe symptoms in languages with a transparent orthography, at least in terms of accuracy (Landerl, Wimmer, & Frith, 1997). In opaque orthographies, the mastery of the alphabetic principle provides only part of the key for decoding and many words cannot be sounded out accurately without having access to the stored phonological representation of the whole word. This may lead to the development of multiple recoding strategies that enable the learner to decode at several different grain sizes, supplementing grapheme-phoneme correspondences with the recognition of letter patterns for rimes and attempts at whole-word recognition (Ziegler & Goswami, 2005), demanding the engagement of a wider range of cognitive skills.

Another language characteristic that is believed to play a role in the early reading process is syllabic complexity. More specifically syllabic complexity is thought to affect how readily children become sensitive to the phonological structure of language (Duncan, Colé, Seymour, & Magnan, 2006), a critical prereading skill. Children who speak French, a language regarded as having a relatively simple syllabic structure characterized by a predominance of open syllables, were found to demonstrate more phonological awareness prior to any formal instruction than their syllabically more complex English-speaking counterparts (Duncan et al., 2006). Moreover, the embedding of grapheme-phoneme correspondences in consonant clusters has been suggested to impede the reading acquisition process (Seymour et al., 2003). Sprenger-Charolles and Siegel (1997) found French first-graders to have more problems reading and spelling bi- and trisyllabic pseudo-words with more complex syllabic structures (including CVC and/or CCV syllables) than those with a simple structure (consisting of CV syllables). Clusters are possibly treated as phonological units and are difficult to split into phonemes (Treiman, 1991). Furthermore, the high level of co-articulation in the consonant phonemes in the cluster might exacerbate the problem (Serrano & Defior, 2012). These difficulties might reflect a deficit in phonological awareness resulting in a difficulty in phonemic segmentation of complex syllable structures and consonant clusters.

A number of researchers have suggested that, in addition to sensitivity to phonemes, sensitivity to the morphological structure of a language plays an important role in the reading process (e.g., Casalis & Louis-Alexandre, 2000; Elbro & Arnbak, 1996; for reviews see Mann, 2000, and Nagy, Carlisle, & Goodwin, 2013), and more particularly in reading difficulties (e.g., Ben-Dror, Bentin, & Frost, 1995; Leikin & Hagit, 2006; Lyytinen & Lyytinen, 2004; Schiff & Raveh, 2007). The recognition of familiar morphemes has been shown to facilitate speed and accuracy of reading and the spelling of morphologically more complex words (Carlisle & Stone, 2005). Moreover, in orthographies with an opaque writing system, many phonemic irregularities (e.g., silent letters *condemn* and *bomb*) may be regularities from the morphological perspective (*condemnation, bombardment*), and consequently the morphological structure of words may function like an anchor to the reader (Schiff & Raveh, 2007). In languages in which the morphological structure of a given word hardly ever changes depending on its function in the sentence or the phrase it belongs to, a word that has been stored in the lexicon will be retrieved with little effort. However, in agglutinative languages such as Finnish, the morphological system results in words of considerable length that contain multiple parts of semantic information. This stacking of functional morphemes to the stem may obscure the stem of the word. Furthermore, given that, at least in Finnish, many root forms are affected by inflection, the ability to recognize roots is not always sufficient to recognize words (Aro, 2004).

Hence, there is more to reading than decoding grapheme-phoneme correspondences only; orthographic transparency, syllabic complexity and morphological complexity all relate to the word recognition process and to each other. The review of the literature included searches of Web of Science, PubMed, PsychInfo, Google Scholar, and various online sources. Our search terms pertained to orthographic transparency, morphological complexity, and syllabic complexity in relation to reading acquisition, and dyslexia.

## Orthographic transparency

In languages with a transparent orthographic system, orthography reflects surface phonology with a high level of consistency. In Finnish, Italian, or Indonesian, for example, a given letter of the alphabet is almost always pronounced the same way irrespective of the word it appears in (e.g., Aro, 2004; Winskel & Lee, 2013; Ziegler et al., 2010). In opaque orthographies, such as English and Danish, however, spelling-to-sound correspondences can be very ambiguous (e.g., Frost, 2012; Seymour et al., 2003). Orthographic transparency expresses in a feedforward, grapheme-to-phoneme fashion and a feedback, phoneme-to-grapheme fashion (Lété, Peereman, & Fayol, 2008). There is general consensus about the approximate classification of several languages in terms of their orthographic transparency (e.g., Seymour et al., 2003). Considering orthographic transparency as a continuum, one can be certain about its extreme positions (e.g., the regular Finnish orthography at one extreme and the irregular English orthography at the other), even though the objective location of each orthography on this transparency continuum may remain uncertain (Aro, 2004). Yet, relatively little quantitative cross-linguistic research has been conducted regarding this matter. Three measures of orthographic transparency, namely regularity, consistency, and entropy, will be discussed in the following sections.

### Regularity approach

The regularity approach assumes there are *regular* mappings governed by symbolic transcription rules and *irregular* mappings that violate these rules. Words that are pronounced regularly, like *cat* /kæt/, or *hint* /hInt/, are read faster than words that are pronounced irregularly, such as *aisle* /aIl/, or *yacht* /jɑt/. This so-called regularity effect has been demonstrated in many studies investigating the role of spelling-to-sound transparency in visual word recognition (Borgwaldt, Hellwig, & De Groot, 2005). The degree of irregularity of any alphabetically written language can be determined once a set of language-specific grapheme-phoneme correspondence rules (GPC rules) has been formulated. In cases in which the mapping deviates from one-to-one, for example when a single grapheme can have multiple pronunciations, the most frequent mapping is considered regular and the others irregular. Regular words are words whose pronunciation or spelling is correctly produced by the grapheme-phoneme correspondence rules of the language, while the pronunciation or spelling of irregular or ‘exception’ words cannot be predicted from these rules (Protopapas & Vlahou, 2009). Regularity of pronunciation or spelling is thus conceptualized as a categorical distinction (Zevin & Seidenberg, 2006). The degree of regularity of the orthography as a whole is then defined as the percentage of words of which the pronunciation agrees with the lexical pronunciation of the whole word according to the GPC rules (Ziegler, Perry, & Coltheart, 2000).

Ziegler et al. (2000) compared the degree of regularity of German and English by examining the percentage of correct rule applications in the two languages assuming that the higher the number of rules that yielded correct results, the more regular the orthography-phonology mapping would be. These numbers were calculated by comparing the pronunciations of monosyllabic words produced by the non-lexical reading route of the dual-route cascaded (DRC) model (Coltheart, Curtis, Atkins, & Haller, 1993) with the correct pronunciation being derived from the CELEX database.

Like other models assuming dual-processing routes for reading, the DRC model distinguishes between a lexical and a non-lexical route for transforming print to sound, using whole-word orthographic representations and grapheme-phoneme conversion (GPC) rules, respectively, to gain access to the phonological output lexicon (Ziegler et al., 2000). Ziegler et al. included three major types of rules in the DRC model: single-letter, multi-letter, and context-sensitive rules. If the non-lexical route and the CELEX database generated the same pronunciation, the rules used to generate the pronunciation were considered correct, while in the case of two deviating pronunciations all the rules used to elicit the pronunciation were considered incorrect. Applying this rule-based approach, the authors found that, averaged across the three rule types used, German rules were correct 90.4% of the time, compared to 79.3% for the English rules. They additionally determined how many monosyllabic words are irregular in German by exclusively using the non-lexical route to read monosyllabic words. By definition, any word that is pronounced incorrectly by the non-lexical route of the DRC model is irregular since it violates the GPC rules. From the 1448 words that were submitted to the DRC model with its lexical route switched off, 150 were read incorrectly via the non-lexical route. This prompted the authors to conclude that using their specific rule set the irregularity in the orthography-phonology mapping for monosyllabic words in German was 10.3%.

Protopapas and Vlahou (2009) calculated the regularity of Greek at word level as the proportion of words read correctly on the basis of their orthographic representation alone. To do so, the authors used an ordered set of 80 rules that could correctly transcribe their complete text corpus (consisting of types and tokens) based on the word-form letter sequences only, without any additional information. When all phonemes were correctly mapped, the word was considered correct. A number of rules were marked as ‘optional’, as the correct pronunciation, being lexically determined, could not be derived from orthographic or phonological information at the grapheme-phoneme level. The study showed that when optional rules were included in the rule set, the regularity of Greek at word level (by token count) was 92.7% and when the optional rules were excluded this was 95.3%. Finally, when optional rules were allowed to apply optionally, with either outcome counting as correct, the word level regularity estimate reached 97.3%.

### Consistency approach

In contrast to the regularity approach, the consistency approach does without the notion of rules with consistency referring to the degree of variability in the correspondences between the orthographic and phonological units of a language (Protopapas & Vlahou, 2009). Consistency computations can be dichotomous or graded and can be performed at the grapheme-phoneme level or at larger grain sizes. In dichotomous analysis, a word or smaller sized unit is regarded consistent when there is only one possible mapping and inconsistent when there are alternative mappings. In graded analyses, the measure of consistency quantifies ambiguity by taking into account the relative frequency of alternative mappings. Here, the level of consistency is expressed as the proportion of dominant mappings over the total number of occurrences of the base unit analyzed. Thus, the consistency of the phoneme /b/ in Spanish is computed as the proportion of words in which the phoneme /b/ occurs with a particular spelling ‘b’, relative to the total number of words that include that phoneme (spelled as ‘b’ or ‘v’). Consequently, the resulting consistency ratio ranges from zero, minimal consistency, to one in case of maximal consistency (Lété et al., 2008).

Feedforward (spelling-to-sound) and feedback consistencies (sound-to-spelling) can vary independently. Using a dichotomous classification at a rime-body level, Ziegler, Jacobs, and Stone (1996) and Ziegler, Stone, and Jacobs (1997) performed both feedforward and feedback analyses of French and English monosyllabic, mono-morphemic words where they considered a word to be consistent when there was a one-to-one correspondence between the spelling body and the phonological body. They found French and English to have quite similar levels of inconsistency in the sound-to-spelling direction, whereas French vowels were much more consistent than the English ones in the spelling-to-sound direction. From the spelling or feedback perspective, 79.1% of the French words and 72.3% of the English words were inconsistent. From the reading or feedforward point of view, 12.4% of the French words and 30.7% of the English words were identified as inconsistent.

In their 2009 study, Protopapas and Vlahou also calculated the consistency of grapheme-phoneme mappings in Greek using a corpus composed of types and tokens. Division of the token sum of the most frequent grapheme for each phoneme by the total number of grapheme-phoneme pairs in the corpus resulted in a ratio of 0.803. To the extent that this outcome can be considered a single-number estimate of the consistency of phoneme-to-grapheme mappings, the Greek orthography is 80.3% consistent in the feedback direction. Using a similar calculation to estimate the consistency of grapheme-to-phoneme mappings, the authors found a 95.1% consistency in the feedforward direction. Using single letters instead of graphemes, the calculation in the reading (feedforward) direction resulted in a substantially lower consistency estimate of 80.3% and a greater number of mappings (173 vs. 118). When stress diacritics were ignored and stressed and unstressed letters (and phonemes) treated as similar, this yielded 88 grapheme-phoneme mappings with an estimated overall token consistency of 96.0% in the feedforward direction and 80.8% in the feedback direction.

### Entropy approach

A third way to measure orthographic transparency is the entropy approach, an index that not only discriminates between cases with many and few alternatives but also between nondominant mappings with substantial and negligible proportions. Entropy is an information-theoretic concept introduced by Shannon (1948) to describe the redundancy of a communication system. In our context, entropy quantifies ambiguity in the prediction of grapheme-to-phoneme mappings and vice versa (Borgwaldt et al., 2005; Protopapas & Vlahou, 2009). The general idea behind the entropy approach is as follows: if a given grapheme (or phoneme) always corresponds to one specific phoneme (or grapheme), the mapping is completely predictable and the corresponding entropy value is zero. The more alternative pronunciations (or spellings) a grapheme (or phoneme) has, the less predictable the mapping becomes and the higher its entropy value will be. Expressing (un)ambiguity in these mappings as entropy values will therefore result in continuous variables, starting at zero for totally unambiguous mappings and increasing with higher degrees of uncertainty. In addition to the number of different pronunciations (or spellings), the relative frequency of these alternative mappings contributes to the entropy value. If there is one dominant grapheme-phoneme correspondence and some of those alternative mappings only occur very seldom, the entropy value will be lower than when all pronunciations occur with approximately the same frequency, resulting in a rather minimal impact of exceptional alternative mappings.

For any unit of orthographic (or phonological) representation *x* that maps onto *n* phonological (or orthographic) alternatives with a probability of *p*_*i*_ for the *i*th alternative, its entropy (*H*) value is calculated as the negative sum over the probability of each separate value of *x* multiplied by the base-2 logarithm of its probability:

$$ {\text{H}} = - \sum\limits_{i = 1}^{n} {p_{i} \log_{2}  p_{i} .} $$

If an orthographic (or phonological) representation *x* always maps onto one single phonological (or orthographic) counterpart, its entropy value equals 0. If *x* has *n* > 1 different mappings, the entropy value’s upper limit is log_2_ *n*. This upper limit is reached when the probabilities of all orthographic (or phonological) representations are the same. Hence, the more alternative mappings the orthographic (or phonological) representation has and the more equiprobable these mappings are, the higher the entropy value will be.

Protopapas and Vlahou (2009) provide an example in Greek in which the phoneme /g/ can be spelled as either <γκ> (85.5%) or <γγ> (14.5%). The phoneme /ç/ can be spelled as <χ> (85.0%), <οι> (7.0%), <ι> (6.9%), or <ει>, <χι>, <χει>, and other combinations with a very low probability of less than 1% each. The entropy value of the phoneme /g/ would be −[0.855 × log_2_ (0.855) + 0.145 × log_2_ (0.145)] = 0.597. Inserting the probabilities for each mapping into the entropy formula, each probability is first multiplied by the binary logarithm (log_2_) of this probability after which the negative sum is calculated, resulting in an entropy value for /g/ of 0.597. If the probabilities for both pronunciations had been the same, the entropy value’s upper limit of 1 (*n* = 2; log_2_2 = 1) would have been reached. The present, considerably lower entropy value results from the fact that /g/ has one truly dominant grapheme <γκ> and one much less dominant one <γγ>. When calculated the same way for the phoneme /ç/, the entropy value equals 0.827.

Seeking to rank English, Dutch, German, French, and Hungarian on the opaqueness-transparency continuum, Borgwaldt, Hellwig, and De Groot (2004) computed entropy values for word-initial letter-to-phoneme correspondences for words in each language. An advantage of concentrating on a word’s initial part is that, rather than the commonly investigated monosyllabic vocabulary, all words in a language can be entered into the analysis. Comparing word-initial letter-to-phoneme correspondences, Borgwaldt et al. (2004) found English to have the most ambiguous orthography, followed by, in descending order, German, French, and Dutch, with Hungarian having the most predictable orthography of all languages analyzed. In terms of phoneme-to-letter mappings, again English had the most ambiguous orthography, with French, German, Dutch, and Hungarian featuring increasingly fewer ambiguities.

In their 2005 study, Borgwaldt et al. added Italian and Portuguese to their analysis while calculating word-initial letter-phoneme entropy values for lemmas instead of words. The authors investigated the relative contributions of vowels and consonants to the overall orthographic transparency and analyzed the influence of entropy values as predictors of reaction times in naming tasks. None of the orthographies studied were found to display completely unambiguous mappings between letters and sounds. In terms of overall spelling-to-sound correspondences analyzed at the word-initial letter-phoneme level, English had the most ambiguous orthography, followed by French, German, Portuguese, Dutch, Italian, and Hungarian. When consonant and vowel letters were analyzed separately, the pattern changed slightly: considering vowels, English remained the language with the most ambiguous letter-to-phoneme correspondences, followed by German, Dutch, French, Portuguese, Italian, and Hungarian, with the latter language showing completely unambiguous vowel-letter/vowel-phoneme mappings. Looking at consonants, the most ambiguous letter-to-sound correspondences were found in French, followed by English, German, Hungarian, Italian, Dutch, and Portuguese. The authors argue that the onset entropy calculations not only inform one of a language’s overall orthographic transparency, but also allow us to rank single words according to the degree of spelling-to-sound ambiguity of their word-initial letters, a variable that was found to correlate significantly with naming latencies. As their analyses revealed, the seven languages showed different characteristics in terms of consonant-vowel ambiguity, which in turn might explain language-specific phonological encoding behavior during the reading process. The authors stipulate that the ambiguity of letter-phoneme mappings should therefore not be ignored in favor of an exclusive focus on larger grain sizes like morphemes.

## Morphological complexity

A large number of the words we read every day are morphologically complex. In French and English this concerns about 75 and 85% of the words, respectively (Grainger & Ziegler, 2011). Morphologically complex words like *work* may, for example, have prefixed and suffixed derivations (e.g., *rework*, *worker*), inflected forms (e.g., *works*, *working*, *workers*), and compounds (e.g., *workplace*).

A common method of quantifying linguistic complexity is to count the occurrences of a variety of hand-picked, intuitively justified properties or complexity ‘indicators’ (Shosted, 2006). As regards morphological complexity, Bane (2008) proposes the number of possible inflections in a ‘typical’ sentence, the number of inflectional categories, and the number of morpheme types as likely indicators. According to Kettunen (2014), the number of cases (e.g., the nominative and accusative case forms) in a language is already indicative of its morphological complexity. Thus, Finnish has fourteen different morphological cases where English has a mere two, implying that Finnish has a large variation in noun forms while English has little. Compounding may also add to the level of morphological complexity. Other morphological categories, such as marking of definiteness and expression of number in the language, are also considered key factors (Stump, 2001).

There have been several suggestions on how to quantify the morphological complexity of languages, but none of the definitions or qualifications proposed has been widely accepted (Kettunen, 2014). A number of these suggestions will be discussed in the following sections.

### Linguistica

Bane (2008) proposes the use of Linguistica^1^ software to approximate the morphological complexity of languages. The software deduces a linguistic system’s morphology from a given text sample based on the ‘minimum description length’ (MDL; Rissanen, 1984), a method of approximating Kolmogorov complexity. The general idea behind this quantification method is that one object is more complex than another insofar that it takes longer to describe, which, in our case, depends on the length of the shortest description of the string in question (see Bane, 2008; Kolmogorov, 1965, for more details). The models (descriptions) that the Linguistica software constructs are lexica consisting of stems, affixes, and ‘signatures’ (Goldsmith, 2001), where signatures describe the possible distributions of affixes upon stems. Bane (2008) provides some example entries in a lexicon derived by Linguistica from a corpus of standard French:

**Table:** 

	Stem	Suffixal signature
(a)	Accompli	Ø.e.t.r.s.ssent.ssez
(b)	Académi	cien.e.es.que
(c)	Académicien	Ø.s

Entry (a) indicates that the stem *accompli*- can take the suffixes -Ø (masculine past particle), -*e* (feminine past particle), -*t* (third person singular), -*r* (infinitive), -*s* (second person singular), -*ssent* (third person plural), and -*ssez* (second person plural). Accordingly, the signature Ø.e.t.r.s.ssent.ssez corresponds to something like the inflectional category “verbs in -*ir*” in French. Likewise, entry (b) signifies that *académi*- is the stem of words like *académicien* (‘academician’), *académie* (‘academy’), *academies* (‘academies’), and *académique* (‘academic’), while entry (c) shows that the word *académicien* is also a stem itself that can take singular -Ø and plural -*s.*

The aggregate complexity of a lexicon is distributed among its stems, affixes, and signatures. In a language with few and simple inflections, most of the information encoded by the lexicon, and hence its complexity, will be present in the set of potential stems. By contrast, for a morphologically more complex language, the Linguistica software will allocate more of that information to the set of potential affixes and signatures. For each stem, affix, and signature, a description length (DL) is calculated and tracked, and the ‘simplest’ model in this case is that with the smallest total description length over all stems, affixes, and signatures. The following morphological complexity metric is proposed in Bane’s study (2008):

$$ {\text{Morphological}}\,{\text{complexity}} = \frac{DL\,({\text{Affixes}}) + DL\,({\text{Signatures}})}{{DL\,({\text{Affixes}}) + DL\,({\text{Signatures}}) + DL\,({\text{Stems}})}} $$

The morphological complexity of a language is then expressed as the sum of the description lengths of affixes and signatures, divided by the lexicon’s total description length. According to Bane, these descriptions lengths are approximations, or indices, of complexity so that the lexicon’s total description length is an approximation of its complexity. Bane proposes that one could also express morphological complexity as a unitless ratio of description lengths (instead of the sum), protecting the metric from the incidental deficiencies of available corpora, which will not generally exhibit the full spectrum of factual morphological combinations in a language.

To explore the empirical behavior of the metric proposed, Bane (2008) selected a total of twenty languages for preliminary appraisal while taking care to include a number of creole languages in order to test whether the proposed metric reflects the often intuitively claimed relative simplicity of their morphology. From each corpus (i.e., Bible translations for fourteen languages and corpora obtained from the Internet for the remaining six creole and pidgin languages), the Linguistica software computed a morphological lexicon of stems, prefixes, suffixes, and signatures describing their possible combinations. Together with their description lengths, the output yielded sufficient information to calculate the morphological complexity ratio. According to the metric, together with Vietnamese, creole and pidgin yielded relatively low morphological complexity values. The remaining languages were ranked plausibly Bane claimed, with Latin (35.5%) and Hungarian (34.0%) being placed the highest in the complexity spectrum, followed by Italian (28.3%), Spanish (27.5%), Icelandic (26.5%), French (23.1%), Danish (22.9%), Swedish (21.9%), German (20.4%), Dutch (19.6%), and English (16.9%).

### Juola method

Juola (1998, 2008) offers an alternative, somewhat simpler approach. He proposes to approximate the Kolmogorov complexity (Kolmogorov, 1965) twice, i.e., before and after a corpus has been deformed in such a way as to efface any morphological information from the text. The ratio of these ‘before’ and ‘after’ measurements reflects the language’s morphological complexity. During the effacement, each word-type is replaced by a random number, thus obscuring its original, inherent linguistic information while retaining external (presumably syntactic) information about the ordering and collocations of the word’s tokens. After distortion, the data is compressed using a compression algorithm with the size of the compressed original word data file then being divided by the size of the compressed distorted word-data file.

Both evaluating Maori, English, Dutch, and French, Juola (1998) and Bane (2008) (the latter using Linguistica) obtained similar relative rankings for these four languages. The Juola method has also been applied by Sadeniemi, Kettunen, Lindh-Knuutila, and Honkela (2008) to 21 EU languages. In line with their expectations, Italian, English, Irish, French, Portuguese, and Spanish generated low complexity values, while the morphologically more complex languages of Finnish, Hungarian, and Polish were ranked at the other end of the scale. The authors do note that some of the results were unexpected: Dutch, Swedish, Danish, and German also yielded quite high values, with those for German even ranking at the top of the scale. Sadeniemi et al. propose that both compound words and the legal genre of the text they used might have boosted the complexity figures here. By contrast, Slovenian, Slovak, Latvian, Czech, and Estonian were expected to rank higher on the complexity scale than they were using this metric.

### Type-token ratio

Type-token ratio (TTR), i.e., vocabulary size divided by text length, is a simple measure of lexical diversity. The basic problem with TTR is that it is affected by the length of the text sample, but Kettunen (2014) has shown that the simple TTR and its more elaborate moving-average type-token ratio (MATTR; Covington & McFall, 2010) can be used for the approximation of morphological complexity of languages. The Finnish researcher computed TTRs by dividing the number of word-form types (i.e., unique string forms) in each selected text by the number of running word forms of the tokenized text and computed MATTR by choosing a window (e.g., 500 words) and then calculating the TTRs for words 1–500, then 2–501, 3–502, and so on to the end of the text. He then used the mean of all resultant TTRs as a measure of lexical diversity of the entire text, which was not affected by text length or by any statistical assumptions. A large variation in unique string forms that contain multiple parts of semantic information (e.g., *work*, *worker*, *working*), would result in a higher type-token ratio and hence according to Kettunen, in a higher morphological complexity. Kettunen analyzed two different sources: a legal text from the EU constitution in 21 languages (Sadeniemi et al., 2008) and non-parallel random data in 16 of the same languages taken from the Leipzig Corpus^2^ that contains a more general genre of texts. Token figures were generated by the MATTR software, whereas types were counted in sorted word files without duplicate forms.

Kettunen compared the TTRs and MATTRs with complexity figures obtained with the Juola method (1998, 2008) and used mean figures of calculations and the number of noun forms in each language as a comparison when discussing the results of each measure. Their results showed that for the EU-constitution data (taken from Kettunen, Sadeniemi, Lindh-Knuutila, & Honkela, 2006), TTR, MATTR and Juola complexity figures correlated moderately (0.049 and 0.041 respectively, *p* < 0.005). No Juola complexity figures were available for the Leipzig data. Figures showed that TTRs and MATTRs correlated highly with each other in both corpora (0.097 for the EU-constitution and 0.093 for the Leipzig corpus figures; *p* < 0.001). English was the least morphologically complex language according to all three measures, followed by Spanish and French, which were ranked among the five least complex languages again by all three measures. Finnish was found to be the most morphologically complex language whereas the Juola method placed Danish above Finnish. Kettunen argues that TTR, MATTR, and Juola’s complexity index all order the languages investigated quite meaningfully based on morphological complexity insofar that all three measures at least grouped most of the languages with the same kind of languages while clearly separating the most and least complex languages.

## Syllabic complexity

The definitions to describe syllabic complexity vary among researchers. Fenk-Oczlon and Fenk (2008) define the concept as the number of phonemes per syllable. Their definition is narrower than the one proposed by Adsett and Marchand (2010), who define syllabic complexity as a measure of how difficult it is, on average, to determine the syllable boundaries in words in a specific language. In Seymour et al. (2003), the syllabic complexity dimension refers principally to the distinction between the Germanic languages, which have numerous closed CVC syllables and complex initial or final consonant clusters and the Romance languages, which have a predominance of open CV syllables with few consonant clusters in both onset and coda position. Thus, there is little consensus on how the syllabic complexity of languages should be determined. Previous research in this field generally adopted one of two approaches, the structural or the behavioral approach. Adsett and Marchand (2010) propose an alternative means, namely syllabification by analogy. All three interpretations will be discussed in the following sections.

### Structural approach

The structural approach uses the frequency and variety of permitted syllables within languages to determine whether they are stress- or syllable-timed (Adsett & Marchand, 2010). In a language spoken with stress-timed rhythm there is considerable variation in syllable length, while, on average, the interval between consecutive, stressed syllables is fairly constant. In a language spoken with a syllable-timed rhythm, on the other hand, the syllable lengths tend to be equal. Adopting this structural approach, Dauer (1983) showed that languages that were more syllable-timed had more CV syllables composed of a single consonant followed by a single vowel. Thus, syllable-timed languages were found to have more open syllables, having no consonants following the vowel portion of the syllable, than closed syllables, where the closed syllables have at least one consonant following the vowel portion of the syllable. In the more stress-timed languages, among which English, Dauer conversely recorded fewer CV syllables and a greater variety in syllable types. Several researchers investigated the CV-syllable frequency of several languages using the following formula:

$$ {\hbox{CV\,frequency}} = \frac{{\left| {\hbox{CV\,syllables}} \right|}}{{\left| {\text{syllables}} \right|}} $$

Adsett and Marchand (2010) observed that this resulted in CV frequency scores of 60.7% for Italian (Bortolini, 1976), 59.0% for European Portuguese (Frota & Vigário, 2001), 58.0% for Spanish (Dauer, 1983), 46.7% for French (Laks, 1995), 43.0% for Dutch (Levelt & Van de Vijver, 1998) and 34.0% for English (Dauer, 1983).

Ramus, Nespor, and Mehler (1999) proposed the duration of vocalic and consonantal intervals (also referred to as *timing*; Arvaniti & Rodriquez, 2013) as indicators of a language’s syllabic structure and complexity. The authors defined vocalic intervals as those segments of speech in which vowels were pronounced and consonantal intervals as those containing the consonant sounds. Analyzing recorded sentences, they found a higher standard deviation of consonant intervals across a sentence to indicate a greater variety in the number of consonants and their overall duration in the syllable, while, similarly, a lower proportion of vocalic intervals reflected a lower frequency of vowels in syllables and therefore the presence of more consonants. Both measures showed that a greater variety in syllable structures is related to a greater number and a greater variety of consonants and thus a higher level of syllabic complexity. Dutch, English, and Polish were highlighted as having a more complex syllable structure as they were found to be more stress-timed languages, while Catalan, French, Italian, and Spanish appeared more syllable-timed, having a simpler syllabic structure according to Ramus et al.

A critical note is required here. Despite the enduring popularity of the view that languages belong to distinct rhythm classes and that these differences are encoded in timing, others have argued strongly against this (e.g., Arvaniti, 2012; Arvaniti & Rodriquez, 2013; Horton & Arvaniti, 2013; Loukina, Kochanski, Rosner, Keane, & Shih, 2011; Tilsen & Arvaniti, 2013), claiming that existing results that support this view are open to other and at least equally plausible interpretations, such as differences in speaking rate or fundamental frequency (F0).

### Behavioral approach

In a study by Seymour et al. (2003), an attempt was made to show the possible effects of syllabic complexity on reading acquisition through analysis of reading-acquisition performance in 13 European orthographies. Based on the results of a large international collaboration between researchers reviewing the characteristics of European orthographies which were thought to likely affect reading acquisition (Niessen, Frith, Reitsma, & Öhngren, 2000), Seymour et al. proposes to classify the orthographies included on the dimensions of syllabic complexity and orthographic depth. According to Seymour et al., the syllabic-complexity dimension distinguishes between Germanic (e.g., German, Danish, and English) and Romance (e.g., French, Italian, and Spanish) languages. Whereas Germanic languages have numerous closed CVC syllables and complex consonant clusters in both onset and coda positions, the Romance type languages have a predominance of open CV syllables with few initial or final consonant clusters. Seymour and colleagues hypothesized that the effort required to acquire literacy increases from simple to complex syllable structures (and from shallow to deep orthographies).

Seymour et al. recorded significantly lower error rates during pseudo-word reading in first- and second-grade children whose native language was Finnish, French, Greek, Italian, Spanish, or Portuguese, all languages that Seymour et al. perceived as having simple syllable structures. Error rates for the children learning to read Austrian, Danish, Dutch, English, Germanic, Icelandic, Norwegian, and Swedish, were greater; languages they considered to be syllabically more complex. A limitation of the study, however, is that the effect of suggested differences in syllabic complexity across languages on reading acquisition cannot be isolated from other differences such as orthographic depth.

### Syllabification by analogy (SbA)

In their 2010 article, Adsett and Marchand propose an alternative, computational way to measure relative syllabic complexity. According to the authors, their automatic syllabification algorithm provides a natural ranking of the complexity of syllabification for each language entered, whereby difficulty to automatically syllabify a language signifies increased complexity. They based their data-driven algorithm on the PRONOUNCE algorithm (Dedina & Nusbaum, 1991) to convert letters to phonemes, which system had then been substantially extended, refined, and adapted for the syllabification-by-analogy (SbA) task (e.g., Marchand & Damper, 2000, 2007). Both non-syllable and syllable-boundary junctures (i.e., a position between two contiguous letters in a word) were marked explicitly, using a * for non-syllable boundaries and a $ for syllable boundaries. The entry *syl*-*la*-*ble*, for example, would be stored as s*y*l$l*a$b*l*e.

Analyzing the syllabic complexity of Basque, Dutch, English, French, Frisian (primarily spoken in the Dutch province of Friesland), German, Italian, Norwegian, and Spanish using their SbA approach, Adsett and Marchand (2010) employed same-sized subsets with matching word length for spelling and pronunciation to facilitate comparisons across languages (see Adsett & Marchand, 2010, for more information about the lexicons used). To verify the representativeness of these lexicons for each specific language, they computed the frequencies of the CV syllables and compared these to values reported in the literature (Bortolini, 1976; Dauer, 1983; Frota & Vigário, 2001; Laks, 1995; Levelt & Van de Vijver, 1998)

Word accuracy was quantified as the number of words syllabified by the method in exactly the same way as was given in the lexicon for that language. All languages were syllabified with an above 85% accuracy for spelling and a 90% accuracy for pronunciation. The results in the pronunciation domain were overall higher than those achieved in the spelling domain, leading the authors to suggest that the SbA method captures the pronunciation dimension best.

Based on the SbA method and with regard to syllabic simplicity in the spelling domain (feedback direction), Spanish came first, followed by Basque, French, Italian, German, Dutch, English, and Norwegian. Accuracy results for Frisian were only analyzed in the pronunciation domain (feedforward direction), in which the languages were ranked, once more from simple to complex, as follows: Spanish, Basque, Italian, French, German, English, Dutch, and Frisian. No pronunciation results were listed for Norwegian.

## Discussion

The specific characteristics of the orthography shape the phonological, orthographic, and morphological processes acquired, essential for fast and efficient word recognition. In this paper, several metrics were discussed that have been devised to quantify orthographic transparency, syllabic complexity, and morphological complexity of alphabetic languages. Based on the current status quo of metrics presented in this paper, it remains difficult to give a clear judgement on which metric seems most valuable for future use in this domain. Besides the fact that relatively little quantitative cross-linguistic research has been conducted regarding these matters and more research is needed before any of the ideas advanced so far will be widely accepted, the best measure also depends on the specific research question and particular orthographies and granularity studied. The use of Linguistica software (Bane, 2008), for example, will only be useful when a difference in the number and complexity of the inflections is expected between the orthographies analyzed. In the light of instruction and intervention development, the ranking of languages proposed by these metrics should be supported by more behavioral data showing differences in reading acquisition and skilled reading between the orthographies studied, a field which for morphological and syllabic complexity measures remains relatively unexplored.

With regard to syllabic complexity measures, the results of the data-driven automatic syllabification algorithm (SbA) by Adsett and Marchand (2010) were in line with previous work applying a structural (Ramus et al., 1999) and behavioral approach (Seymour et al., 2003) and resulted in similar distinctions between orthographies. When comparing Adsett and Marchand’s SbA-pronunciation results for Dutch, English, French, Italian, and Spanish with the speech results Ramus et al. (1999) had obtained for these languages, lower word accuracies were obtained in the pronunciation domain for the languages judged by Ramus et al. to have a more complex syllabic structure (Dutch and English) than those believed to be syllabically less complex (French, Italian, and Spanish). As to spelling, Adsett and Marchand arrived at the same conclusion as Seymour et al. (2003), with their SbA approach having yielded higher word-accuracy values in the spelling domain for Italian, French, and Spanish than for the four Germanic languages.

Although promising, more research is needed to increase the value of these metrics. One general difficulty is the variety in definitions. To make predictions about whether, and if so how, any of these orthographic aspects might affect reading acquisition and skilled reading, one cannot go without a clear and widely accepted definition of the specific aspect studied. Schmalz, Marinus, Coltheart, and Castles (2015) recently tried to tackle this issue for orthographic depth in their review by trying to get to the bottom of what is meant with orthographic depth in different studies and by proposing their definition based on theories of reading and previous research in this domain. Having a widely used definition of orthographic transparency, syllabic complexity and morphological complexity, will facilitate cross-linguistic studies addressing these notions and different researchers replicating these studies in other orthographies.

Compared to syllabic complexity, the orthographic transparency measures have received much more attention and researchers have been trying to challenge and improve the orthographic transparency measures over the past decades. Moreover, Borgwaldt et al.’s (2005) onset-entropy measure has also been used in large-scale behavioral studies of cross-linguistic differences (Landerl et al., 2013; Moll et al., 2014; Vaessen et al., 2010; Ziegler et al., 2010). A number of limitations and proposed refinements are discussed below. The regularity and consistency studies using mono-syllabic words (Ziegler et al., 1996, 1997, 2000) stumble on difficulties to fully represent the whole writing system and all its complexities to which the reader is exposed. Moreover, cross-linguistic differences in the proportion and representativeness of monosyllabic words with respect to the full spectrum of mappings cannot be excluded (Protopapas & Vlahou, 2009). This monosyllabic bias present in the DRC model for example, is eliminated by focusing on the first letter only as is done by Borgwaldt et al. (2004, 2005) using onset-entropy values. This also increases the comparability between orthographies as all words in all orthographies have initial letters. Nonetheless, while entropy, in contrast to the consistency and regularity approach, is able to discriminate between cases with many and few alternative mappings, and between non-dominant mappings with substantial and insignificant proportions, word-initial entropy values such as used by Borgwaldt et al., may still fail to represent the full spectrum of potential mapping complexities in different parts of the word. In English, for example, vowels occur more frequent in the middle of the word and it is often the vowel pronunciation that is unpredictable (Treiman, Mullennix, Bijeljac-Babic, & Richmond-Welty, 1995). Moreover, in French, the spelling-to-sound irregularities mostly occur in the final consonants, which are often silent (Lété et al., 2008). In Dutch, the words *kiezen* (to choose) and *[ik] kies* ([I] choose), have different spellings despite being forms of the same verb. This is because the ‘z’ in *kiezen* is pronounced as /z/, whereas the final phoneme in *kies* is pronounced as /s/ due to devoicing of final consonants in Dutch. In this example, the phoneme /s/ is represented by the grapheme ‘s’, and morphological transparency is sacrificed for phonological transparency (Landerl & Reitsma, 2005). By contrast, in the case of *krabben* (to scratch) and *[ik] krab* ([I] scratch), the devoiced final consonant ‘b’ is pronounced as /p/ in /kʀɑp/, but is still written as ‘b’. Here phonological transparency is sacrificed for morphological transparency. In both examples, onset-entropy would possibly result in an overestimation of the orthographic transparency. Surprisingly, to our knowledge no study whatsoever has been conducted on the use of coda-entropy values. Protopapas and Vlahou (2009) show that the use of word-initial single-letter mappings results in a substantial underestimation of the orthographic transparency of Greek when compared to whole-word across-letter calculations. Another possibility would be to use word-form databases to assess languages in their natural reflected form (Hofmann, Stenneken, Conrad, & Jacobs, 2007; Protopapas & Vlahou, 2009), instead of lemmas such as used by Borgwaldt et al. (2005). Word-form databases include morphological variations of the same lemma, such as *work*, *worked*, *working*, whereas lemma databases merely contain the ‘base’ form *work*. Moreover, frequency of occurrence is among the strongest predictors of how fast a word can be recognized or read aloud (Balota, Yap, & Cortese, 2006). Protopapas and Vlahou endorse transparency measurements in terms of token counts, using word forms weighted by the number of their occurrences in a representative text or speech corpus. A more conservative method would be to consider both type- and token-frequency counts, as recommended by Hofmann et al. (2007).

Despite the limitations, the studies discussed in the present paper do trigger our thoughts about the complexity or simplicity of languages. From the perspective of orthographic transparency, English can be considered a complex language, whereas Finnish may be perceived as simple. Studies have suggested that with regard to reading acquisition, transparent orthographies with high grapheme-phoneme consistency are more easily acquired than opaque and complex writing systems featuring a large number of inconsistent and irregular spellings (Aro & Wimmer, 2003; Seymour et al., 2003). However, when it comes to word-recognition, characteristics of the Finnish morphology reduce the effectiveness of these ‘beneficial’ factors since the majority of Finnish words are polysyllabic and tend to be long due to the highly productive compounding, a rich derivational system, and agglutinative morphology (Aro, 2004; Lyytinen et al., 2006). English scores the lowest on the morphological complexity measures TTR, MATTR, and Juola (Kettunen, 2014) among all languages included, whereas Finnish was found to be the most (or second most) morphologically complex language. Nonetheless, behavioral research has shown that more than 95% of Finnish students acquires accurate reading skills during the first year (Holopainen, Ahonen, & Lyytinen, 2001), while the rate of early reading acquisition was suggested to be slower by a ratio of about 2.5:1 in English than in most European orthographies (Seymour et al., 2003). This suggests that Finnish children acquire efficient strategies to overcome the potential difficulty resulting from the morphological complexity of the Finnish language. It has been argued that Finnish children are highly oriented toward the details of spoken language in order for them to differentiate words with small (single phonemic) variations. This would account for the large number of exceptional inflections that are already understood by Finnish children at school-entry age (Torppa, Lyytinen, Erskine, Eklund, & Lyytinen, 2010). Morphological complexity has been suggested to most likely influence the automatization of reading in Finnish, i.e., how efficiently one learns to use larger units (Leinonen et al., 2001). Reading fluency, rather than accuracy, is seen as the most central factor being compromised among dyslexic readers in transparent orthographies such as Finnish (Lyytinen, Erskine, Hämäläinen, Torppa, & Ronimus, 2015).

For future research, we would suggest for more cross-linguistic studies to be conducted comparing two orthographies which are similar on as many aspects as possible, but different on the particular component of interest. The measures proposed in this study may be used to compare languages on the specific aspect investigated or may provide a starting point for other research focusing on the development of quantitative measures. Knowing that this will be a difficult task, several different studies will need to be conducted on the same set of orthographies, and these studies will need to be replicated in other languages. Proposed rankings need to be supported by behavioral studies of reading acquisition and skilled reading. Furthermore, within-language studies that are able to isolate a particular aspect that has been argued to drive cross-linguistic differences may also provide valuable information. Schiff, Katzir, and Shoshan (2013) for example examined the effects of orthographic transparency on fourth-grade readers of Hebrew, revealing a different pattern of reading development among the children with dyslexia. The Hebrew script consists of both vowelized and unvowelized script. The vowelized script is a highly regular and consistent orthography representing both consonants and vowels using both vowel letters and diacritic marks, whereas the unvowelized script is written without any diacritics representing vowels that are not conveyed by the basic alphabet and is considered orthographically irregular and inconsistent (Schiff, 2012). Interestingly, the authors’ findings suggested that, while the development of reading among Hebrew children typically relied on vowelization for intact acquisition of orthographic representations during early reading, no such reliance was found among the young dyslexic readers. This might be due to the flawed grapheme-phoneme conversions skills of dyslexics, preventing them from using the vowelized script as a self-teaching mechanism for the development of an orthographic lexicon necessary for the later decoding of unvowelized words (Share, 1995).

There is more to reading than sounding out graphemes. In this paper, we gave an overview of measures of orthographic transparency, morphological complexity and syllabic complexity, thereby discussing studies that propose several metrics at various grain sizes at which readers crack the orthographic code to identify written words. Despite our growing insight in these processes, in order to help children and adolescents overcome language and literacy problems, we still need to learn more about orthographic differences and about how to take advantage of language-specific orthographic, syllabic, and morphological sources of information.

## Footnotes

1 Available at http://linguistica.uchicago.edu.

2 Leipzig Corpora Collection Download Page: http://corpora.uni-leibzig.de/download.html.
